# Building Ohmic Contact Interfaces toward Ultrastable Zn Metal Anodes

**DOI:** 10.1002/advs.202102612

**Published:** 2021-10-20

**Authors:** Huanyan Liu, Jian‐Gan Wang, Wei Hua, Huanhuan Sun, Yu Huyan, Shan Tian, Zhidong Hou, Junchang Yang, Chunguang Wei, Feiyu Kang

**Affiliations:** ^1^ State Key Laboratory of Solidification Processing Center for Nano Energy Materials School of Materials Science and Engineering Northwestern Polytechnical University and Shaanxi Joint Lab of Graphene (NPU) Xi'an 710072 China; ^2^ Shenzhen Cubic‐Science Co., Ltd Nanshan District Shenzhen 518052 China; ^3^ Engineering Laboratory for Functionalized Carbon Materials and Shenzhen Key Laboratory for Graphene‐Based Materials Graduate School at Shenzhen Tsinghua University Shenzhen 518055 China

**Keywords:** high performance, metal oxides, work function, zinc anodes, zinc‐ion batteries

## Abstract

Zn metal holds grand promise as the anodes of aqueous batteries for grid‐scale energy storage. However, the rampant zinc dendrite growth and severe surface side reactions significantly impede the commercial implementation. Herein, a universal Zn‐metal oxide Ohmic contact interface model is demonstrated for effectively improving Zn plating/stripping reversibility. The high work function difference between Zn and metal oxides enables the building of an interfacial anti‐blocking layer for dendrite‐free Zn deposition. Moreover, the metal oxide layer can function as a physical barrier to suppress the pernicious side reactions. Consequently, the proof‐of‐concept CeO_2_‐modified Zn anode delivers ultrastable durability of over 1300 h at 0.5–5 mA cm^−2^ and improved Coulombic efficiency, the feasibility of which is also evidenced in MoS_2_//Zn full cells. This study enriches the fundamental comprehension of Ohmic contact interfaces on the Zn deposition, which may shed light on the development of other metal battery anodes.

## Graphical abstract

A universal Zn‐metal oxide Ohmic contact interface model is demonstrated to effectively enable improved Zn^2+^ diffusion kinetics and a reduced Zn nucleation barrier, thus achieving a dendrite‐free and side‐reaction‐free Zn deposition chemistry for significant improvement of the electrochemical performance in both the symmetric cells and full cells.

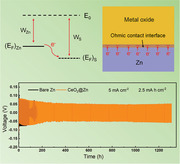

## 1 Introduction

Rechargeable aqueous Zn‐ion batteries have garnered an extensive research interest due to their attractive merits of low price, non‐pollution, and rich abundance in the earth's crust.^[^ ^1^ ^]^ However, the Zn anodes are of particular suffering on uncontrollable Zn dendrite growth and severe side reactions (i.e., corrosion and hydrogen evolution reaction (HER)) upon plating/stripping operations, which significantly hinders the practical application.^[^ ^2^ ^]^ The dendrite formation may pierce the separator and eventually renders battery failure or even safety hazards. In addition, the continuous accumulation of irreversible by‐products (e.g., Zn_4_SO_4_(OH)_6_·*x*H_2_O and H_2_) not only reduces the Coulombic efficiency (CE) of the cell, but also increases the inner pressure that may inflate the cell to failure.^[^ ^3^ ^]^ Therefore, solving the dendrite and side reaction issues are of great significance for the commercialization of Zn anodes.

To date, a variety of design strategies, such as surface modification,^[^ ^4^ ^]^ electrolyte modulation,^[^ ^5^ ^]^ and a three‐dimensional (3D) design,^[^ ^6^ ^]^ have been developed to deal with the above‐mentioned critical problems. In particular, surface modification attracts tremendous attention due to its remarkable protective effect and easy manipulation. The recent endeavors are primarily concentrated on metals,^[^ ^7^ ^]^ carbon matrix,^[^ ^8^ ^]^ polymers,^[^ ^9^ ^]^ metal‐organic frameworks,^[^ ^10^ ^]^ and metal oxide coatings.^[^ ^11^ ^]^ Among these candidates, metal oxides show a great potential to effectively suppress the dendrite growth on the Zn anode, which will propel the commercialization of aqueous Zn‐ion batteries. For instance, ZrO_2_ featuring a high dielectric constant and good chemical stability was demonstrated as an excellent artificial coating layer that provides more nucleation sites and enhanced Zn^2+^ transport kinetics through “space charge polarization”, which guarantees the uniform Zn stripping/plating and long cycling stability.^[^ ^12^ ^]^ An ultrathin Al_2_O_3_ layer was coated on a Zn anode to enable enhanced corrosion resistance and suppressed dendrite.^[^ ^13^ ^]^ TiO_2_ coating was also revealed to be capable of suppressing the corrosion and HER of Zn anode.^[^ ^14^ ^]^ More recently, Wang et al. revealed that TiO_2_ with exposed (001) facet exhibits relatively low Zn affinity, thereafter preventing the vertical growth of Zn dendrites and stabilizing the Zn‐electrolyte interface.^[^ ^15^ ^]^ Despite these great endeavors, however, the underlying mechanism on why and how these metal oxides affect the Zn deposition still remains unclear.

**Figure 1:**
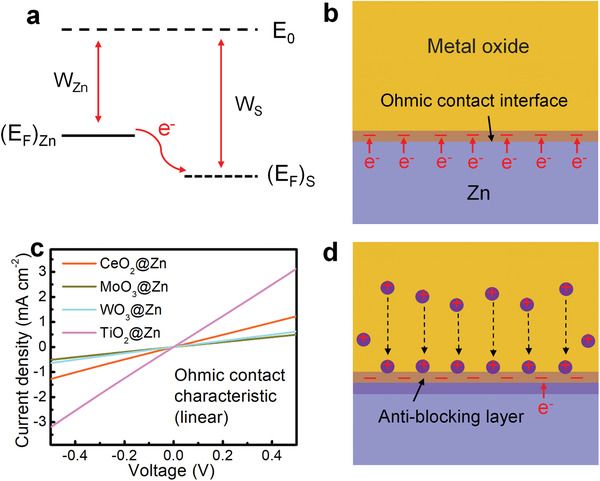
a) Schematic of electrons flowing from metallic Zn to a metal oxide semiconductor, here, *W*_Zn_ and *W*_S_ represent the work functions of Zn and semiconducting oxides. (*E*_F_)_Zn_ and (*E*_F_)_S_ represent the Fermi levels of metallic Zn and semiconducting oxides, and *E*_0_ is the vacuum level. Schematic illustration of b) the formation of an Ohmic contact interface between Zn and metal oxides, and d) the corresponding uniform Zn plating process upon cycling. c) Current–voltage curves of the Zn anodes modified by CeO_2_, TiO_2_, WO_3_, and MoO_3_ layers.

It is important to notice that metallic Zn owns lower work function (3.6–3.8 eV)^[^ ^16^ ^]^ than many metal oxide semiconducting materials, such as TiO_2_ (4.4–5.0 eV),^[^ ^17^ ^]^ WO_3_ (4.3–4.8 eV),^[^ ^18^ ^]^ MoO_3_ (6.2–6.7 eV),^[^ ^19^ ^]^ and CeO_2_ (4.3–4.7 eV).^[^ ^20^ ^]^ The high work function difference could drive electrons flowing from metallic Zn to the oxide semiconductors, thus building an Ohmic contact interface (**Figures** **1**a and b). The negative charges accumulated at the interface can attract the cations to diffuse inward (Figure 1d). Inspired by these characteristics, in this work, we propose a universal Zn‐metal oxide Ohmic contact interface model to homogenizing Zn deposition. As a proof of concept, mesoporous CeO_2_ was employed as the coating layer on the Zn anode. Impressively, the CeO_2_ layer enables improved Zn^2+^ diffusion kinetics and reduced Zn nucleation barrier, thus rendering a dendrite‐free Zn deposition chemistry. Meanwhile, the corrosion and HER are effectively inhibited on account of the protective role of CeO_2_ layer. As a result, the CeO_2_‐coated Zn (CeO_2_@Zn) electrode exhibits a long lifespan up to 1300 h with high CE, and moreover, a full cell based on CeO_2_@Zn//MoS_2_ also delivers a brilliant rate capability and long cycling stability.

## 2 Results and Discussion

Figure 1a schematically present the work functions or Fermi energy levels of metallic Zn and semiconducting metal oxides. The energy band difference propels the electron movement from metallic Zn to the interfaced metal oxides until an equilibrium state,^[^ ^21^ ^]^ forming a typical Ohmic contact interface from a physics viewpoint (Figure 1b). Current–voltage curves were performed to certify the contact type built at the Zn‐metal oxide interfaces. As shown in Figure 1c, all the Zn anodes modified by the listed metal oxides (i.e., CeO_2_, TiO_2_, WO_3_, and MoO_3_) distinctly exhibit linear features, which reveals the non‐rectifying Ohmic contact characteristics.^[^ ^22^ ^]^ It should be noted that the negative charge accumulation will lead to the establishment of an electron enrichment region at the interfaced metal oxides, which is called an “anti‐blocking layer” herein.^[^ ^23^ ^]^ When immersed into the electrolyte, the negative charges in the anti‐blocking layer will have crucial effects in attracting the cations but repelling the anions through electrostatic interaction, thus regulating the cation flux for homogeneous Zn deposition (Figure 1d).

**Figure 2:**
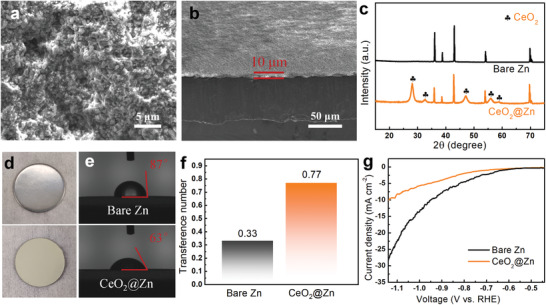
a) Top‐view and b) cross‐section SEM images of CeO_2_@Zn. c) XRD patterns and d) optical images of bare Zn and CeO_2_@Zn. e) Contact angles on bare Zn and CeO_2_@Zn. f) Zn^2+^ transference number from the symmetrical cells with and without CeO_2_ layer. g) LSV curves of bare Zn and CeO_2_@Zn in 1 m aqueous Na_2_SO_4_ electrolyte at a scan rate of 5 mV s^−1^.

To demonstrate the practicality of this unique Zn‐metal oxide interface model, as a proof‐of‐concept, mesoporous CeO_2_ was cast‐coated to the Zn anode as the protective layer (see the Experimental section). Scanning electron microscopy (SEM) image reveals the nanoparticle morphology of CeO_2_ (**Figure** **2**a). The longitudinal observation macroscopically confirms the flat surface of the CeO_2_ coating with a thickness of ≈10 µm (Figure 2b; Figure S1, Supporting Information). The phase structure of CeO_2_ was examined by X‐ray diffraction (XRD) technique. As shown in Figure 2c, the distinct diffraction peaks centered at 28.6°, 33.1°, 47.6°, 56.4°, and 59.2° can be readily assigned to the (111), (200), (220), (311), and (222) crystal facets of cubic CeO_2_ (PDF: 03‐065‐5923), respectively.^[^ ^24^ ^]^

Figure 2d presents the optical images of bare Zn and CeO_2_@Zn anodes. Notably, the pristine Zn exhibits a metallic luster character, while the surface of CeO_2_@Zn is slight yellow stemming from the intrinsic color of nano‐CeO_2_. Contact angle measurements were performed to compare the hydrophilia of the two samples. Impressively, the CeO_2_@Zn electrode delivers dramatically improved electrolyte wettability (63°) compared to the pristine Zn (87°), (Figure 2e). The enhancement of the hydrophilicity is more obvious from the observation of dynamic measurement for 30 min (Figure S2, Supporting Information). N_2_ adsorption/desorption isotherm was then carried out to analyze the porosity of CeO_2_. As shown in Figure S3, Supporting Information, the type IV isotherm curve suggests the mesoporous nature of CeO_2_ with a high specific surface area of 138 m^2^ g^−1^. The corresponding pore size distribution manifests that the pore width is clustered at 5–10 nm, which could afford numerous nanosized channels for fast Zn^2+^ transport and thus homogeneous Zn^2+^ deposition. In addition, it has been reported that the coating layer with high electrical resistance results in a high potential gradient through “space charge polarization” to facilitate the diffusion of Zn^2+^ through the coating layer.^[^ ^25^ ^]^ In order to demonstrate the inferior electronic conductivity of CeO_2_ layer, voltage–time curves were used to evaluate the electrical resistance (Figure S4, Supporting Information). Encouragingly, the value of CeO_2_ was estimated as high as 4.97 × 10^4^ Ω cm, corresponding to a poor electronic conductivity of 2.01 × 10^−5^ S cm^−1^ (details see the Supporting Information). The synergistic combinations of good wettability, sufficient sub‐nano transportation channels, and high potential gradient are of significant benefit for improving the Zn^2+^ diffusion capability.

The Zn^2+^ transference number (*t*_Zn2+_) was then applied to quantitatively estimate the Zn^2+^ diffusion capability of CeO_2_ layer. Here, the Bruce–Vincent method was executed to evaluate the $t_{Zn^{2+}}$ value in the system (Figure S5, Supporting Information):^[^ ^26^ ^]^

(1) $$t_{Zn^{2+}}=\frac{I_{S}\left(V-I_{0}R_{0}\right)}{I_{0}\left(V-I_{S}R_{S}\right)}$$

Where *V* is the applied potential (20 mV); *I*_0_ and *R*_0_ are the initial current and interface resistance, respectively; *I*_s_ and *R*_s_ represent the steady‐state current and interface resistance, respectively. As displayed in Figure 2f, the $t_{Zn^{2+}}$ value in the bare Zn system is as low as 0.33, which can be ascribed to the unrestricted migration of SO_4_^2−^ anions compared to hydrated Zn^2+^. In sharp contrast, the value for CeO_2_@Zn symmetric cell dramatically increases to 0.77 due to the improved Zn^2+^ diffusion kinetics and restrained anions transfer, which is highly desirable for high performance battery operation.

To evaluate the effect of CeO_2_ coating layer on suppressing the side reactions, both CeO_2_@Zn and bare Zn electrodes were soaked in 2 m ZnSO_4_ for 10 days. As shown in Figure S6, Supporting Information, the bare Zn suffers severe corrosion with an obvious color evolution from bright to gray. It can be observed that the Zn surface was covered by plenty of hexagonal flakes with a diameter up to 30 µm, which can be identified as the Zn_4_SO_4_(OH)_6_·*x*H_2_O by‐product (Figures S7 and S8, Supporting Information). In strong contrast, there are no visual and structural changes on the surface of soaked CeO_2_@Zn electrode (Figure S9, Supporting Information), and moreover, the signals of Zn_4_SO_4_(OH)_6_·*x*H_2_O species are fundamentally absent (Figure S10, Supporting Information), suggesting the brilliant anti‐corrosion ability. A linear polarization curve was measured to further confirm the excellent anti‐corrosion feature. As shown in Figure S11, Supporting Information, the CeO_2_@Zn symmetrical cell manifests a much smaller corrosion current than the bare Zn one, indicating a larger corrosion resistance. In addition, the HER performance of the bare Zn and CeO_2_@Zn electrodes was experimentally compared using linear sweep voltammetry (LSV) measurements. Remarkably, the CeO_2_@Zn electrode shows a higher HER overpotential of 0.90 V at 5 mA cm^−2^ and a higher Tafel slope of 336 mV dec^−1^, compared to that of the bare Zn (0.78 V and 199 mV dec^−1^) (Figure 2g; Figure S12, Supporting Information). These results demonstrate that the CeO_2_ layer exerts a significant role in inhibiting the side reactions of corrosion and HER.

**Figure 3:**
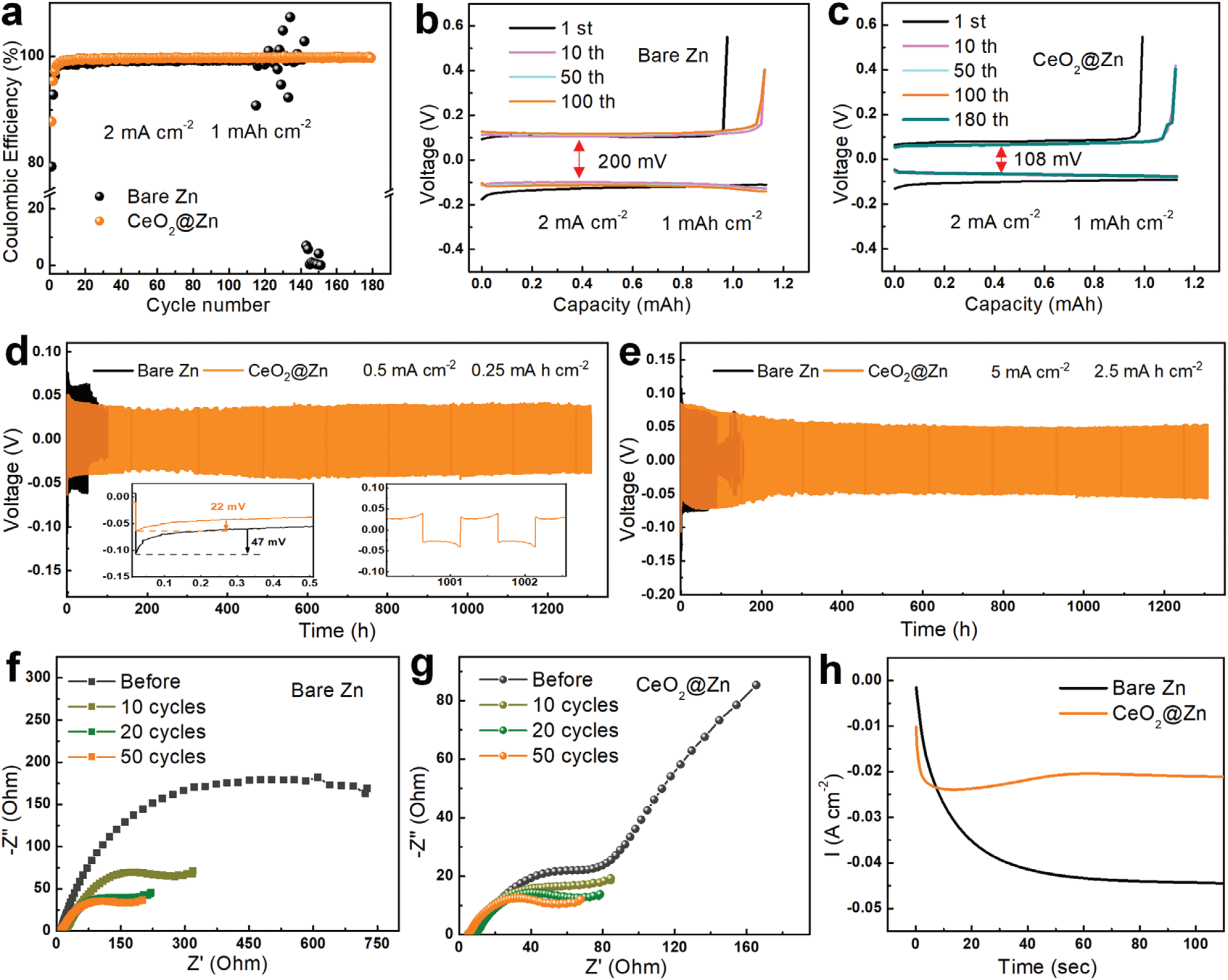
a) Coulombic efficiencies of bare Zn and CeO_2_@Zn plating/stripping on Cu foil at 2 mA cm^−2^, and corresponding galvanostatic cycling curves for b) Zn—Cu and c) CeO_2_@Zn—Cu asymmetry cells at different cycles. Cycling performance of bare Zn and CeO_2_@Zn symmetric cells at d) 0.5 mA cm^−2^ for 0.25 mAh cm^−2^ and e) 5 mA cm^−2^ for 2.5 mAh cm^−2^. EIS plots of f) bare Zn and g) CeO_2_@Zn symmetric cells after different plating/striping cycles. h) Chronoamperometry curves of bare Zn and CeO_2_@Zn at an overpotential of −150 mV.

The electrochemical plating/stripping behavior of Zn was investigated using asymmetric Zn—Cu and CeO_2_@Zn—Cu half cells. As shown in **Figure** **3**a, the CeO_2_@Zn—Cu cell exhibits a high initial CE of 87.8% at 2 mA cm^−2^, which increases quickly to a steady CE of 99.8% over 180 cycles. In sharp contrast, the bare Zn—Cu cell delivers a lower initial CE of 79.4%, and fluctuates to failure after 140 cycles, which can be attributed to the existence of parasitic reactions and Zn dendrites. Figure 3b,c compares the corresponding galvanostatic plating/striping profiles of two cells at specific cycles. Notably, the CeO_2_@Zn—Cu cell manifests a smaller polarization potential of 108 mV, which is almost half of the bare Zn—Cu cell (200 mV). The high and stable CE and lower polarization potential electrochemically elucidates the importance of the CeO_2_ layer on promoting the Zn platting/stripping reversibility and kinetics.

Symmetric cells were assembled to evaluate the cycling stability of CeO_2_@Zn and bare Zn electrodes. Figure 3d illustrates the cycling performance of the two cells measured at 0.5 mA cm^−2^. Impressively, the CeO_2_@Zn cell delivers a long life‐time up to 1300 h, which is far better than that of the bare Zn (<100 h). In addition, the CeO_2_@Zn anode shows a small voltage hysteresis of 42 mV with ultrastable voltage–time curves even after 1000 h (inset). In strong contrast, a high voltage hysteresis of 67 mV and a quick short circuit are observed for bare Zn. The similar phenomenon is further identified at high current densities of 1.0 (Figure S13, Supporting Information) and even at 5.0 mA cm^−2^ (Figure 3e). The comparison of the CE of CeO_2_@Zn and the cycle life of the symmetric cells with the reported Zn anodes further demonstrates the brilliant properties of CeO_2_@Zn (Figure S14, Supporting Information). Such an excellent electrochemical performance can be ascribed to the enhanced plating kinetics of Zn^2+^ and regulated nucleation of Zn^2+^ induced by the CeO_2_ layer. To investigate the Zn^2+^ plating kinetics, the nucleation overpotential of the assembled symmetric cells is given out. As shown in the inset of Figure 3d, the CeO_2_@Zn symmetric cell manifests a much lower nucleation overpotential (22 mV) than that of bare Zn (47 mV) at 0.5 mA cm^−2^, suggesting that the Zn^2+^ nucleation barrier is significantly reduced. Even at high current densities of 1 and 5 mA cm^−2^, a similar trend can also be concluded (Figure S15, Supporting Information). Electrochemical impedance spectroscopy (EIS) was measured to further prove the improved Zn^2+^ plating kinetics. Figures 3f and g depict the EIS data of bare Zn and CeO_2_@Zn after different plating/striping cycles. Importantly, the CeO_2_@Zn symmetric cells exhibit smaller charge‐transfer resistance (*R*_ct_) compared to the bare Zn cells, indicating rapid Zn^2+^ transference stemming from the mesoporous CeO_2_ layer. In order to clarify the effect of polyvinylidene fluoride (PVDF) binder on the Zn anode, we also evaluated the electrochemical behaviors of PVDF‐coated Zn anode. As shown in Figure S16, Supporting Information, the PVDF‐coated Zn anode shows larger voltage polarization and Zn growth overpotential than that of bare Zn, indicating that PVDF layer poses a negligible effect on the improvement of the Zn^2+^ deposition kinetics. Therefore, the enhanced Zn^2+^ deposition kinetics of CeO_2_@Zn originates from CeO_2_ layer instead of PVDF.

Chronoamperometry (CA) measurement was carried out to fundamentally analyze the deposition behavior of Zn^2+^ on the anode surface. As shown in Figure 3h, the current on the bare Zn electrode continuously increases far beyond 100 s when applying an overpotential of −150 mV, indicating a frantic 2D nucleation process due to the planar diffusion of Zn^2+^.^[^ ^9^ ^]^ This nucleation mode will cause the subsequent Zn nucleus to aggregate and grow into Zn dendrites, which is typical of the noted “tip effect”.^[^ ^27^ ^]^ In sharp contrast, the CeO_2_@Zn electrode manifests a typical 3D diffusion and nucleation process as the planar nucleation in a CeO_2_@Zn symmetric cell accounts for only 5 s, suggesting the regulated Zn^2+^ diffusion and nucleation upon deposition.

**Figure 4:**
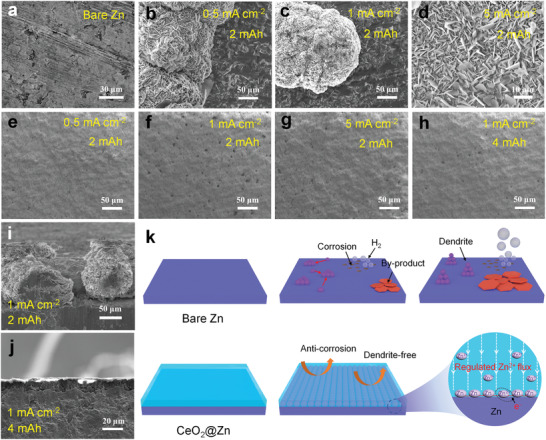
SEM images of a) bare Zn and after plating 2 mAh cm^−2^ at b) 0.5 mA cm^−2^, c) 1 mA cm^−2^, and d) 5 mA cm^−2^. SEM images of CeO_2_@Zn after plating 2 mAh cm^−2^ at e) 0.5 mA cm^−2^, f) 1 mA cm^−2^, and g) 5 mA cm^−2^. h) SEM images of CeO_2_@Zn plating 4 mAh cm^−2^ at 1 mA cm^−2^. Cross‐section SEM images of i) bare Zn and j) CeO_2_@Zn at 1 mA cm^−2^ for 4 mAh cm^−2^. k) Schematic illustration of the Zn^2+^ deposition mechanisms on bare Zn and CeO_2_@Zn.

SEM imaging was used to further investigate the deposition behavior of Zn^2+^ nucleation and growth on bare Zn and CeO_2_@Zn. **Figure** **4**a–d displays the surface morphology of bare Zn and after plating for 2 mAh cm^−2^ at current densities of 0.5, 1, and 5 mA cm^−2^, respectively. After Zn deposition, large protrusions are unevenly distributed on the surface of Zn at 0.5 and 1 mA cm^−2^, which may be due to the rampant 2D diffusion at low current densities that leads to the accumulation of Zn at the initial nucleus, corresponding well with the electrochemical analysis in Figure 3h. The magnified SEM image in Figure S17, Supporting Information, corroborates the existence of microsized dendrites on large protrusions at 1 mA cm^−2^. These large protrusions can reach a size of 100 µm, which can easily pierce the separator and cause the battery short circuit (Figure 4b,c, i). When the current density increases to 5 mA cm^−2^, the large protrusions disappear and the surface is covered by plenty of vertical dendrites with a size of ≈10 µm. This is because that the large current provides enough nucleation energy for nucleation before the planar diffusion of Zn^2+^. However, the stiff and sharp dendrites can also short‐circuit the battery. In comparison, the CeO_2_@Zn electrode shows a completely flat surface at various current densities (Figure 4e–g), manifesting a homogeneous Zn deposition behavior. Even when a higher areal capacity of 4 mAh cm^−2^ is deposited onto the CeO_2_@Zn at 1 mA cm^−2^, the electrode still remains flat surface (Figure 4h). The cross‐section view of the electrodes with a plating capacity of 2 mAh cm^−2^ at 1 mA cm^−2^ elucidates the homogeneous Zn deposition on the CeO_2_@Zn more clearly (Figure 4i,j).

On the basis of the above analysis, the deposition mechanism was schematically illustrated in Figure 4k. Specifically, for the bare Zn, the frantic Zn^2+^ planar diffusion leads to the dendrite formation, and the side reactions downgrade the CE and cycling stability. In strong contrast, benefiting from the CeO_2_ layer and Ohmic contact interface, the deposition of Zn^2+^ presents uniform 3D nucleation and diffusion, thus leading to dendrite‐free Zn anode with excellent cycling performance. Moreover, the reduced side reactions on the CeO_2_@Zn anode are conductive to the high CE and cycling stability. In order to verify the universality of the Ohmic contact interface model, commercial TiO_2_, WO_3_, and MoO_3_ semiconducting materials were examined as the protective layer of Zn (Figure S18, Supporting Information). Impressively, all of the assembled symmetric batteries exhibit outstanding cycling performance at 1 mA cm^−2^ with a plating/stripping capacity of 0.5 mAh cm^−2^ (Figure S19, Supporting Information), convincingly proving the effectiveness of our universal Zn‐metal oxide Ohmic contact interface model.

**Figure 5:**
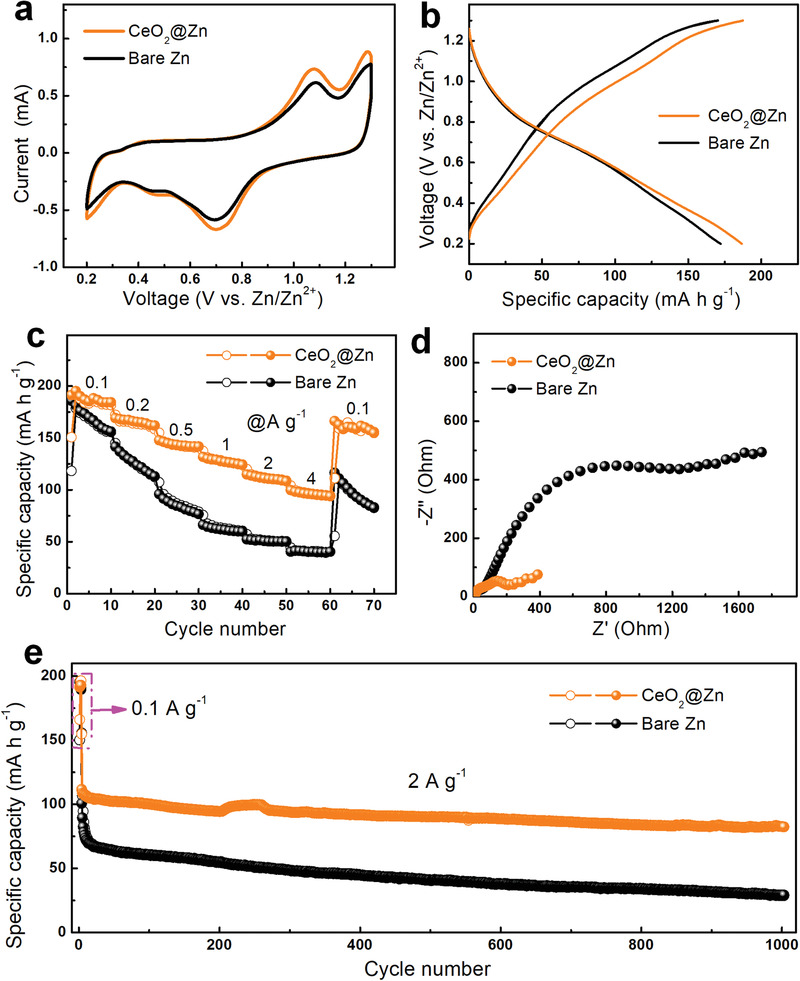
a) *CV* curves (0.1 mV s^−1^) and b) charge/discharge profiles (0.1 A g^−1^) of the bare Zn/MoS_2_ and CeO_2_@Zn/MoS_2_ full cells. Comparison of c) rate performance, d) Nyquist plots, and e) cycling stability of the bare Zn//MoS_2_ and CeO_2_@Zn//MoS_2_ batteries.

Zn//MoS_2_ full cells were assembled to demonstrate the practical application of the CeO_2_@Zn anode (Figure S20, Supporting Information). Cyclic voltammogram (CV) curves (**Figure** **5**a) manifest a typical redox couple at ≈0.7 and ≈1.1 V, which belong to the reversible insertion/extraction of Zn^2+^ into/from the MoS_2_ framework, respectively.^[^ ^28^ ^]^ In addition, the similar CV shape suggests the negligible influence of CeO_2_ layer on the Zn^2+^ insertion chemistry in Zn/MoS_2_ batteries. The galvanostatic charge–discharge profiles of the two batteries agree well with the CV curves (Figure 5b). Figure 5c and Figure S21, Supporting Information, show the rate performance of the two full cells. Notably, the CeO_2_@Zn//MoS_2_ battery delivers an absolutely higher specific capacity of 96 mAh g^−1^ at a high rate of 4 A g^−1^ compared to that of bare Zn//MoS_2_ (47 mAh g^−1^). More importantly, the specific capacity of CeO_2_@Zn//MoS_2_ battery stabilizes at 167 mAh g^−1^ when the current density goes back to 0.1 A g^−1^, while the bare Zn//MoS_2_ cell undergoes dramatic capacity fading. This excellent rate capability can be attributed to the improved Zn^2+^ diffusion kinetics in the CeO_2_@Zn/MoS_2_ battery, as further elucidated by the decreased *R*_ct_ value (Figure 5d; Figure S22, Supporting Information). The cycling performance of the two cells was compared in Figure 5e. Remarkably, the CeO_2_@Zn/MoS_2_ battery maintains a high reversible capacity of 84 mAh g^−1^ at 2 A g^−1^ after 1000 cycles. In sharp contrast, a rapid capacity decrease can be observed in the initial 10 cycles for the control battery, and the capacity eventually drops to only 30 mAh g^−1^ after 1000 cycles. Overall, the CeO_2_@Zn/MoS_2_ cells exhibit both good rate capability and longevity by virtue of the enhanced reversibility and Zn^2+^ diffusion kinetics of CeO_2_‐coated Zn anode.

## 3 Conclusions

In summary, a universal Zn‐metal oxide Ohmic contact interface model has been established to regulate the homogeneous Zn deposition behavior. The as‐demonstrated CeO_2_ coating layer significantly renders improved Zn^2+^ diffusion kinetics and a reduced Zn^2+^ nucleation barrier, therefore achieving dendrite‐free Zn deposition electrochemistry. In combination with the suppressed electrolyte‐induced side reactions, as a consequence, the as‐designed CeO_2_@Zn anode exhibits enhanced Coulombic efficiency, smaller voltage hysteresis, and a splendid cycling lifetime (up to 1300 h under 2.5 mAh cm^−2^@5 mA cm^−2^). When assembled with the MoS_2_ cathodes, the CeO_2_@Zn enables the full cells to manifest a superior rate (96 mAh g^−1^ at 4 A g^−1^) and cycling performance. It is believed that our fundamental findings herein will provide a valuable guideline for developing ultrastable metal anodes beyond Zn‐ion batteries.

## Conflict of Interest

The authors declare no conflict of interest.

## Supporting information

Supporting Information

## Data Availability

Research data are not shared.
