# Supplementary material for: Building Ohmic Contact Interfaces toward Ultrastable Zn Metal Anodes
Source: Adv Sci (Weinh). 2021 Oct 20;8(23):2102612. doi: 10.1002/advs.202102612 (PMC8655195; doi:10.1002/advs.202102612)
Supplement: Supplementary file 1 — Supporting Information [file ADVS-8-2102612-s001.pdf]

WILEY-VCH

© 2021 Wiley-VCH GmbH

ADVANCED SCIENCE
Open Access

Supporting Information

for *Adv. Sci.,* DOI: 10.1002/advs.202102612

Building Ohmic Contact Interfaces toward Ultrastable Zn Metal Anodes

Huanyan Liu, Jian-Gan Wang, Wei Hua, Huanhuan Sun, Yu Huyan, Shan Tian, Zhidong Hou, Junchang Yang, Chunguang Wei,* **and** *Feiyu Kang*

Supporting Information

Building Ohmic Contact Interfaces toward Ultrastable Zn Metal Anodes

Huanyan Liu, Jian-Gan Wang, Wei Hua, Huanhuan Sun, Yu Huyan, Shan Tian, Zhidong Hou, Junchang Yang, Chunguang Wei, Feiyu Kang*

H. Y. Liu, Prof. J.-G. Wang, W. Hua, H. H. Sun, Y. Huyan, S. Tian, Z. D. Hou, Prof. J. C. Yang

State Key Laboratory of Solidification Processing, Center for Nano Energy Materials, School of Materials Science and Engineering, Northwestern Polytechnical University and Shaanxi Joint Lab of Graphene (NPU), Xi'an 710072, China

E-mail: wangjiangan@nwpu.edu.cn (J.-G. Wang)

Dr. C. G. Wei

Shenzhen Cubic-Science Co., Ltd, Nanshan District, Shenzhen 518052, China

Prof. F. Y. Kang

Engineering Laboratory for Functionalized Carbon Materials and Shenzhen Key Laboratory for Graphene-based Materials, Graduate School at Shenzhen, Tsinghua University, Shenzhen 518055, China

Experimental Section

Synthesis of mesoporous CeO_2 nanoparticles: In a typical procedure, 1.8 g cerium nitrate hexahydrate, 3.0 g Pluronic P123, and 1.5 mL nitric acid were mixed in 15 mL 1-butanol at 60 °C to form a homogeneous sol. Then, the sol was transferred to a ventilated oven at 120 °C for 3 h. The products were collected, washed with excess ethanol, and dried under vacuum overnight. The solids were then calcinated at 450 °C for 4 h with a heating rate of 5 °C/min to obtain mesoporous CeO_2.

Synthesis of CeO_2 coated Zn anode (CeO_2@Zn): The CeO_2 coated Zn foils were made up of Zn foils and CeO_2 slurry that was prepared by mixing polyvinylidene fluoride (PVDF) and the above synthesized mesoporous CeO_2 at a mass ratio of 1:9 in N-methyl pyrrolidone (NMP) solution. The resulting slurry was scraped on Zn foils and dried in vacuum at 60°C for 12 h, and thus the designed CeO_2@Zn was completely prepared. The TiO_2, WO_3, and MoO_3 coated Zn anodes were prepared using the same method except CeO_2.

Synthesis of MoS_2 cathode on carbon cloth: In a typical synthesis of MoS_2, 0.076 g ammonium molybdate tetrahydrate and 1 g thiourea were dissolved in 35 ml of deionized water under magnetic stirring. Subsequently, a piece of carbon cloth (3*6 cm^2) was immersed into the above mixture solution, which was then transferred into a 50 ml Teflon-lined autoclave. After a hydrothermal synthesis at 180 °C for 24 h, the MoS_2 grown carbon clothe was obtained. The clothe was used as the cathode directly after being punched into disks (Φ = 12 mm).

Assembly of Zn/Zn symmetric cells and Zn/MoS_2 cells: CR2025-type coin symmetric cells were assembled with identical electrodes of bare Zn or CeO_2@Zn (diameter: 12 mm, thickness: 80 μm), 2 M $ZnSO_4$ electrolyte and glass fiber separators. Zn/MoS_2 full cells were assembled by using pure Zn or CeO_2@Zn as anodes, 2 M $ZnSO_4$ as electrolyte and glass fiber as separators. All batteries were assembled in open air conditions and aged for 12 hours before electrochemical measurements.

Materials characterization: The physical morphology and structure were characterized by scanning electron microscopy (SEM, NanoSEM 450, FEI) and transmission electron microscope (TEM, Talos F200X, FEI). X-ray diffractometer (XRD, X'Pert PRO MPD, Philips) was carried out to confirm the crystallographic structure of the products using Cu-Kα as the radiation source (λ= 1.5418 Å). The surface wettability of anodes was performed by OCA25 contact angle measuring system (Dataphysics, Germany).

Electrochemical measurement: The cycling and rate performance were measured on the Land Battery Testing System at room temperature. Cyclic voltammetry (CV) and electrochemical impedance spectra (EIS) measurements were collected on Solartron electrochemical workstation (1400 + 1470E, England). The specific surface area and the pore size distribution of CeO_2 were analyzed on Micromeritics ASAP 2020 system at liquid N_2 temperature (77 K) based on the Brunauer-Emmett-Teller (BET) method and Barrett-JoynerHalenda (BJH) model.

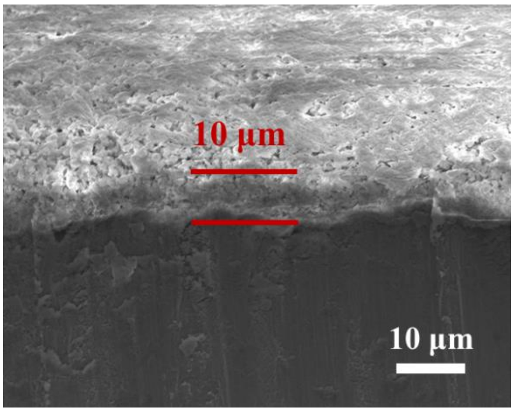

Figure S1 The magnified cross-section SEM image of CeO_2@Zn anode.

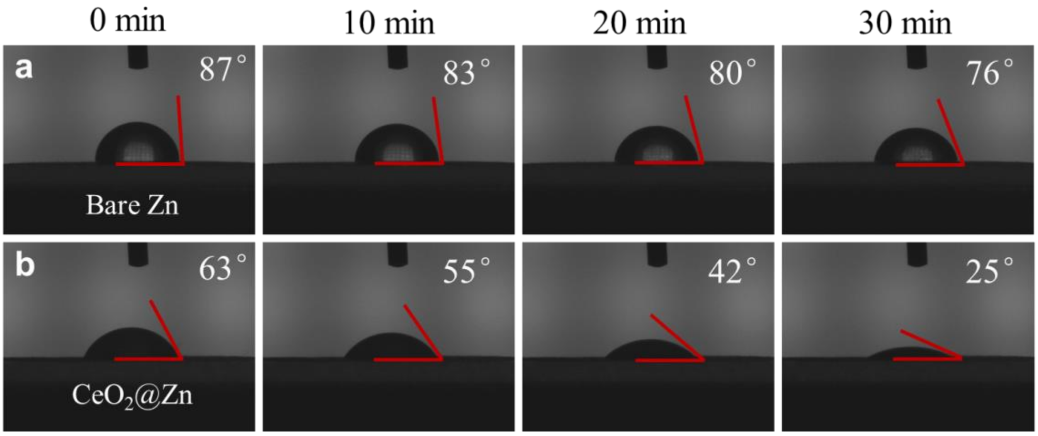

Figure S2 Dynamic contact angles of water on (a) bare Zn and and (b) CeO_2@Zn.

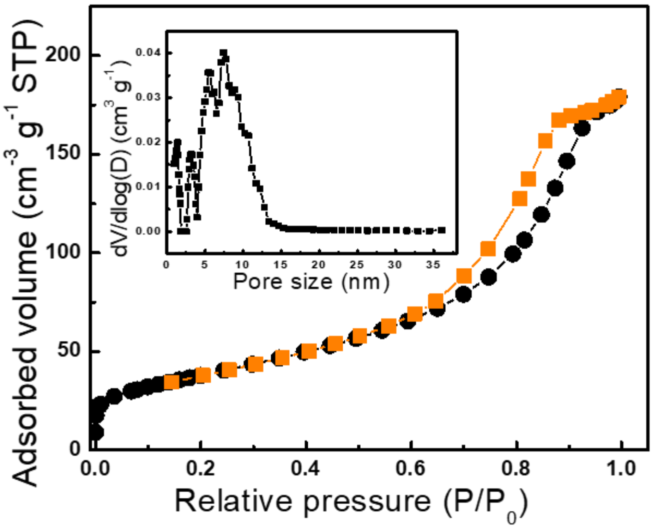

Figure S3 N_2 adsorption/desorption isotherm and pore size distribution (insert) of nano-CeO_2.

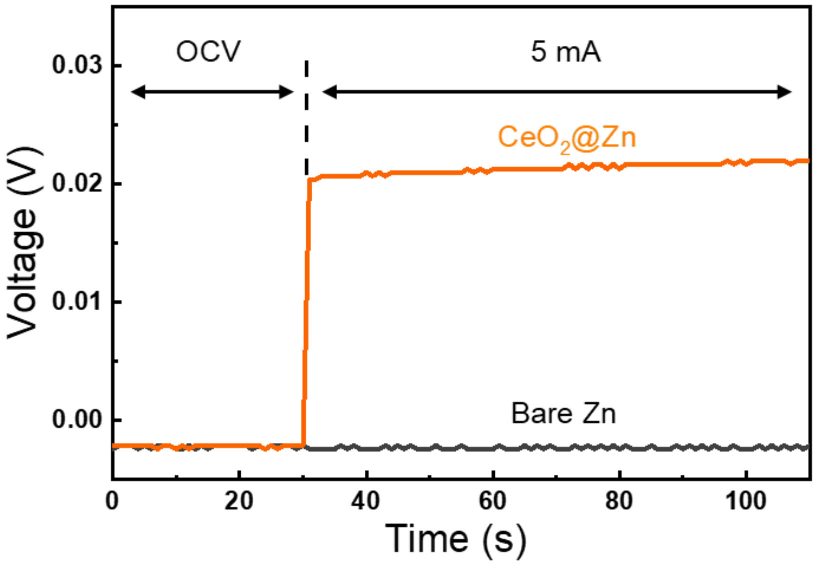

Figure S4 Voltage-time profiles of bare Zn and CeO_2@Zn at open cyclic voltage (OCV) following by a constant current of 5 mA. The bare Zn and CeO_2@Zn are sandwiched between two stainless steel current collectors.

For the resistivity measurement of the CeO_2 layer, the voltage response to a direct current of 5 mA was measured for CeO_2@Zn anode. The electrical resistivity, ρ, of the CeO_2 layer was calculated based on the following equation:

$$\rho = \frac{R*S}{L} = \frac{U*S}{I*L}$$

where L is the thickness of CeO_2 layer, I is the applied current, R is the resistance, S is the contact area between the stainless steel and the CeO_2@Zn anode (1.13 cm^{-2}), and U is the average voltage. The calculated value of the electronic resistivity for the CeO_2 layer is ~4.97×10^4 Ω·cm, corresponding to an electronic conductivity of ~2.01×10^{-5} $S \cdot cm^{-1}$.

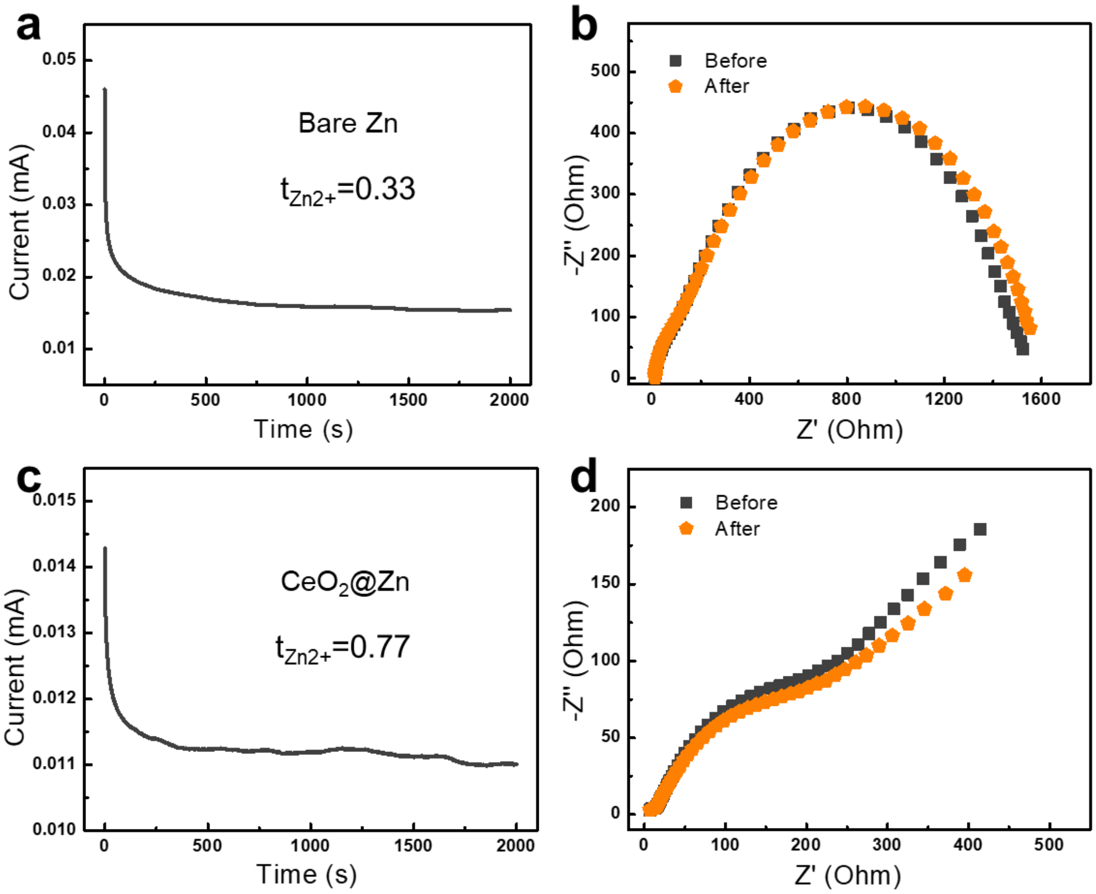

Figure S5 Current variation with time during polarization of (a, b) bare Zn and (c, d) CeO_2@Zn symmetrical batteries at 25 °C with applied potential difference of 20 mV.

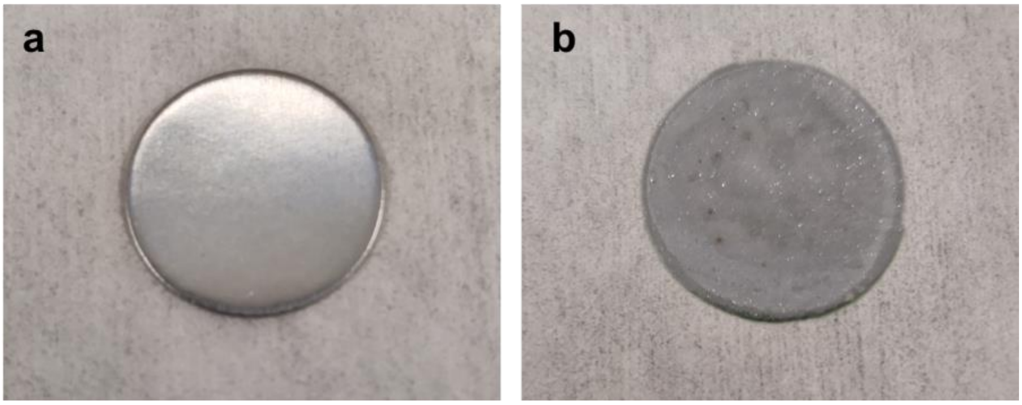

Figure S6 Optical images of bare Zn (a) before and (b) after being immersed in 2M $ZnSO_4$ for 10 days.

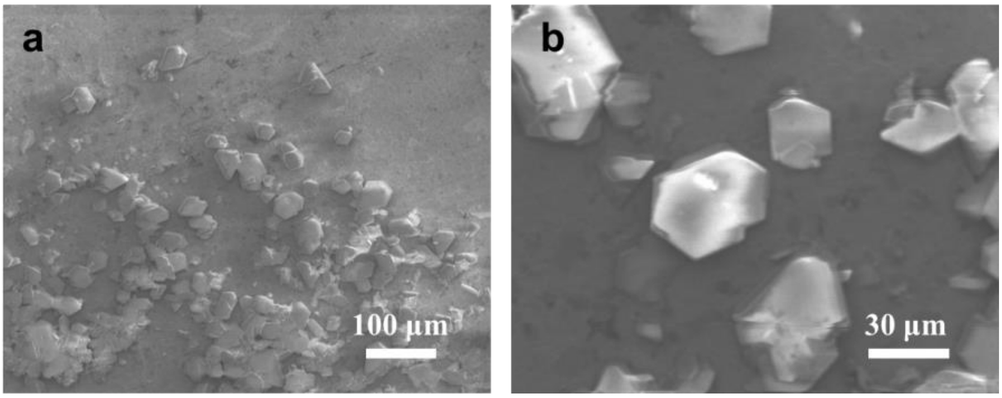

Figure S7 SEM images of bare Zn after being immersed in 2M $ZnSO_4$ for 10 days.

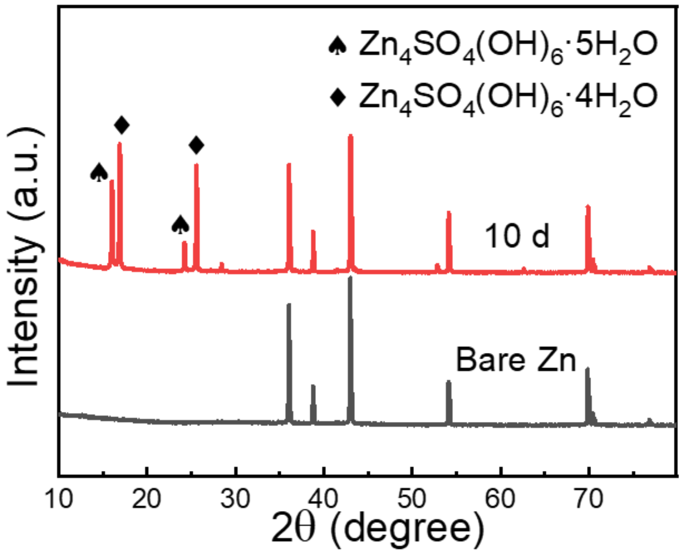

Figure S8 XRD patterns of bare Zn before and after being immersed in 2M $ZnSO_4$ for 10 days.

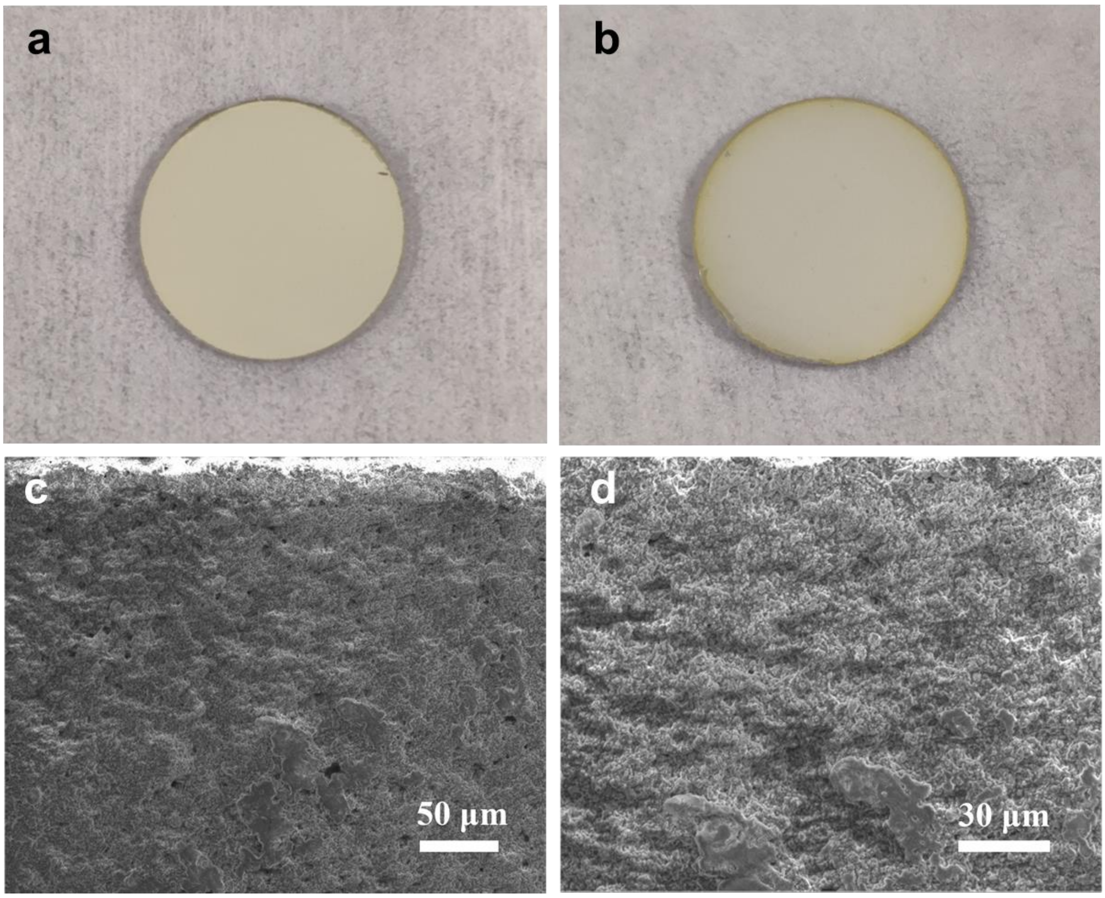

Figure S9 Optical images of CeO_2@Zn (a) before and (b) after being immersed in 2M $ZnSO_4$ for 10 days. (c, d) SEM images of CeO_2@Zn after being immersed in 2M $ZnSO_4$ for 10 days.

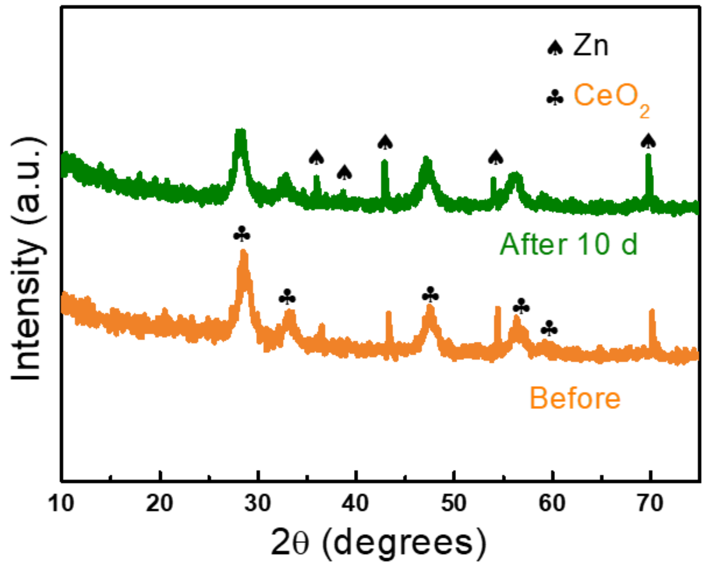

Figure S10 XRD patterns of CeO_2@Zn before and after being immersed in 2M $ZnSO_4$ for 10 days.

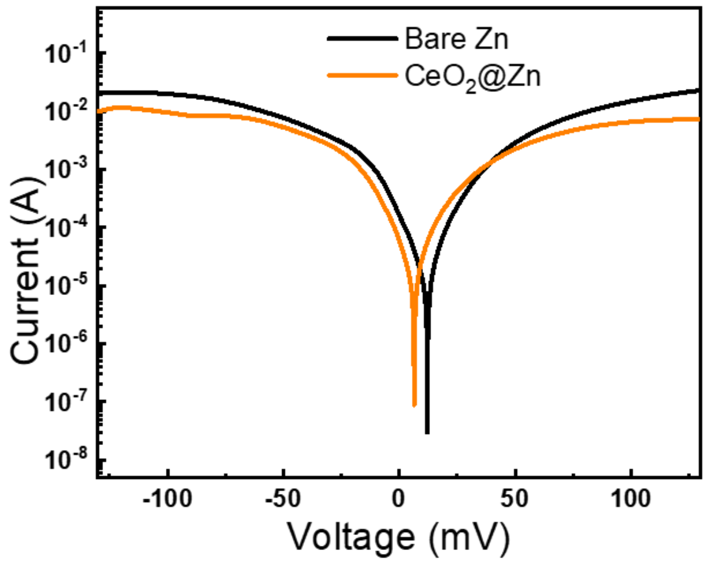

Figure S11 Linear polarization curves of bare Zn and CeO_2@Zn in 2M $ZnSO_4$ electrolyte.

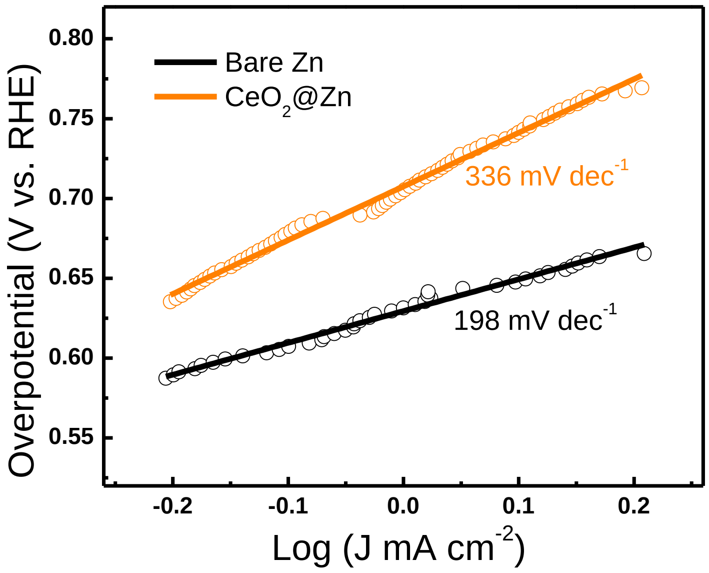

Figure S12 Tafel plots of bare Zn and CeO_2@Zn electrodes for HER.

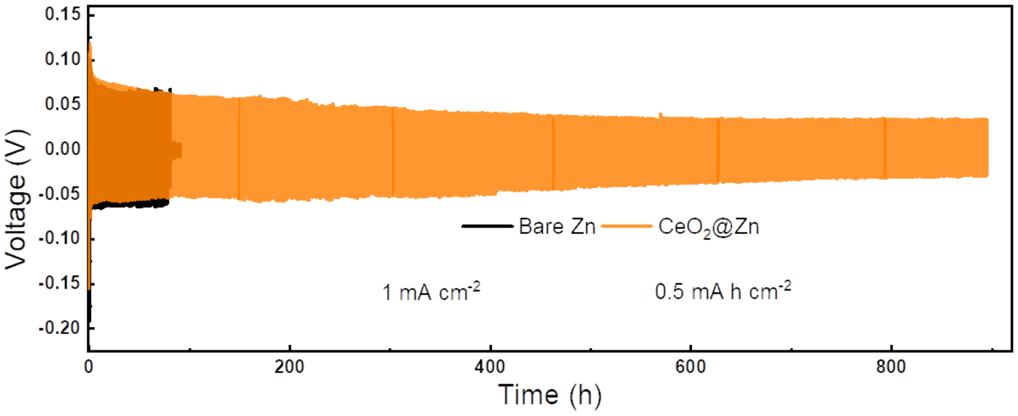

Figure S13 Cycling performance of bare Zn and CeO_2@Zn symmetric cells at 1 mA cm^{-2} for 0.5 mAh cm^{-2}.

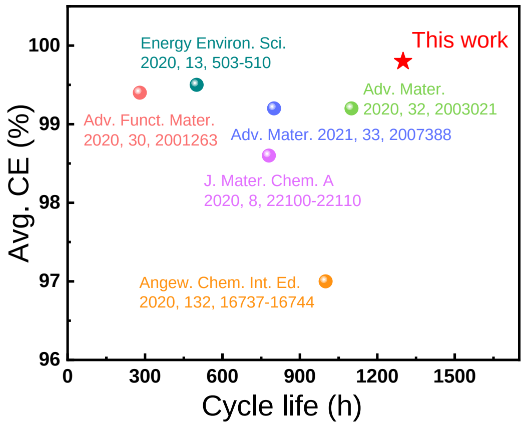

Figure S14 The comparison of the CE of CeO_2@Zn and the cycle life of the symmetric cells at high current densities with the reported Zn anodes.

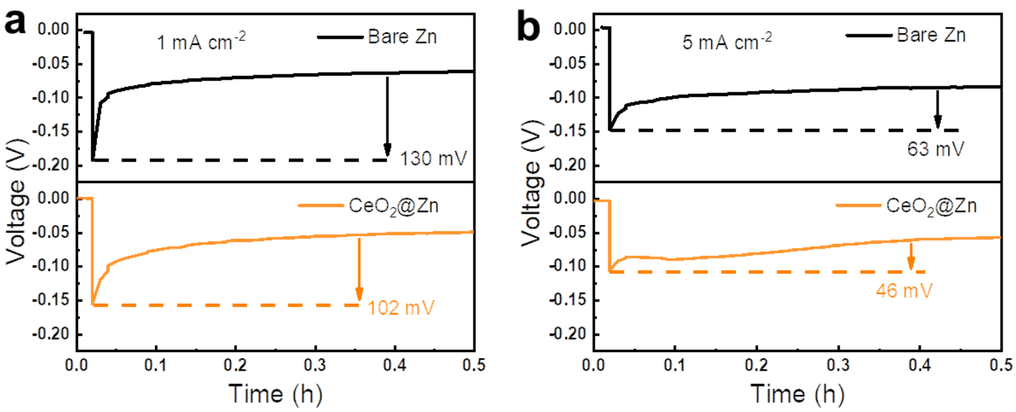

Figure S15 Nucleation overpotential of bare Zn and CeO_2@Zn symmetric cells at (a) 1 mA cm^{-2} and (b) 5 mA cm^{-2}.

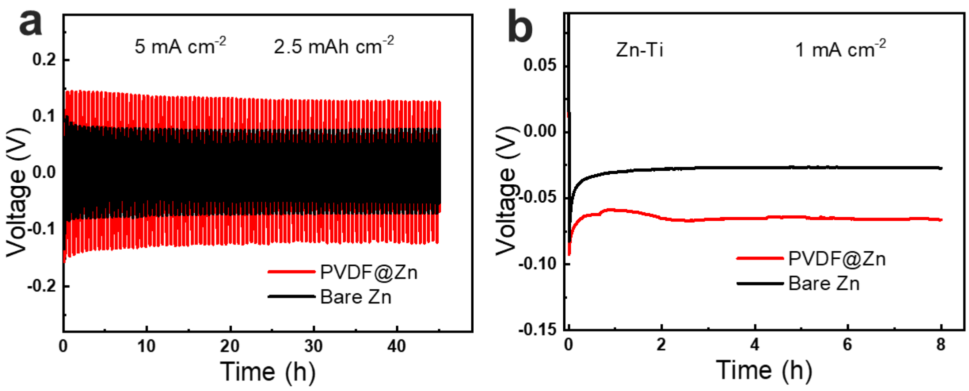

Figure S16 (a) Cycling performance of PVDF@Zn and bare Zn at 5 mA cm^{-2}. (b) Zn deposition curves of PVDF@Zn/Ti and bare Zn/Ti asymmetric cells at 1 mA cm^{-2}.

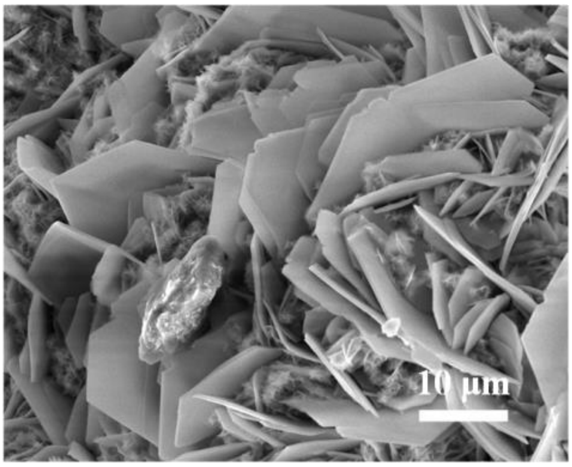

Figure S17 Magnified SEM image of bare Zn after depositing 2 mAh cm^{-2} at 1 mA cm^{-2}.

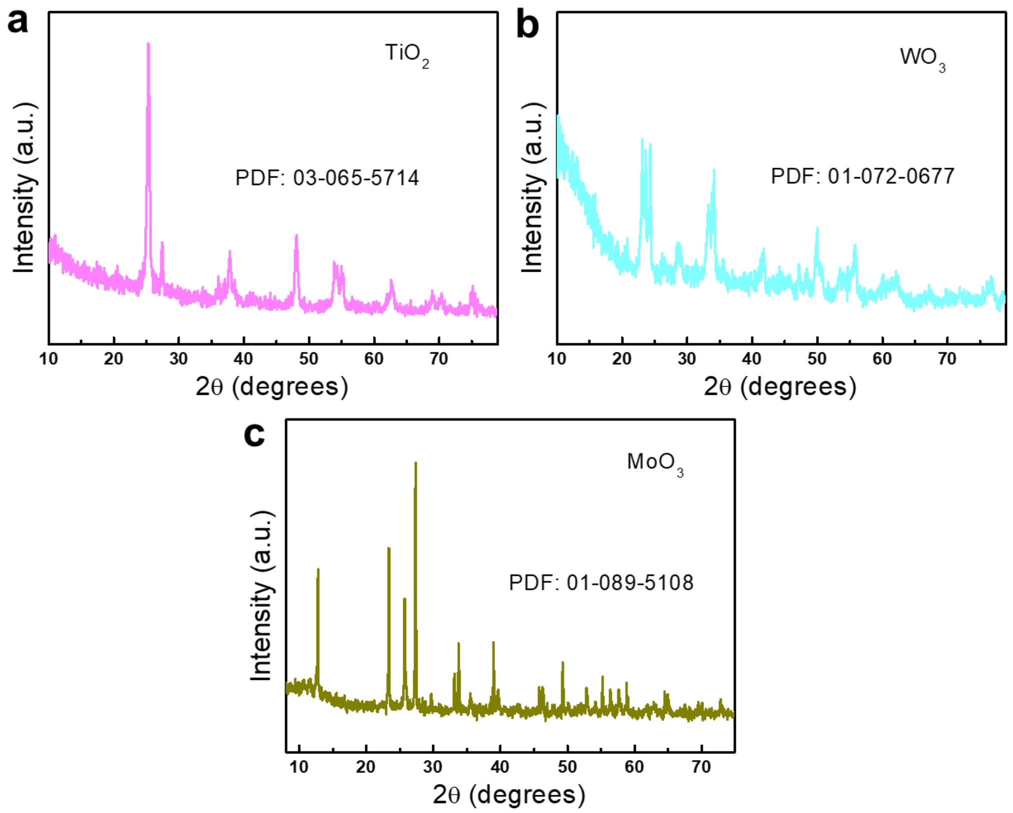

Figure S18 XRD patterns of commercial (a) TiO_2, (b) WO_3, and (c) MoO_3 nanoparticles.

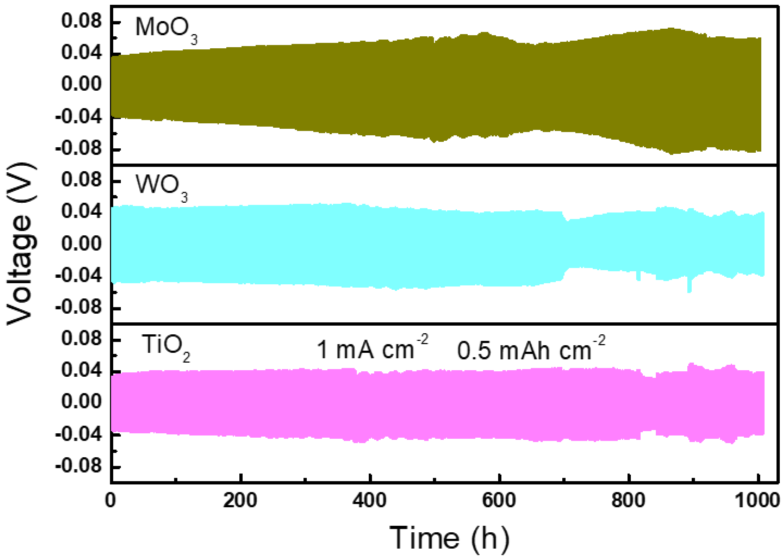

Figure S19 Cycling stability of TiO_2, WO_3, and MoO_3 protected Zn symmetric cells at 1 mA cm^{-2}.

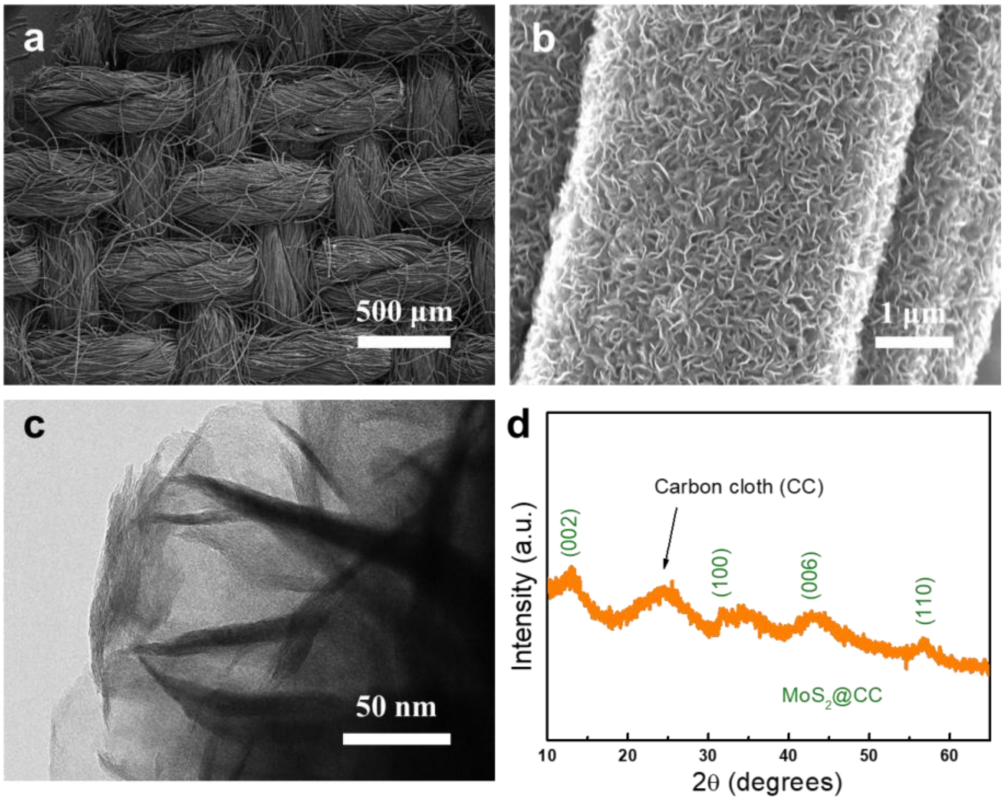

Figure S20 (a, b) SEM, (c) TEM images, and (d) XRD pattern of MoS_2 cathode.

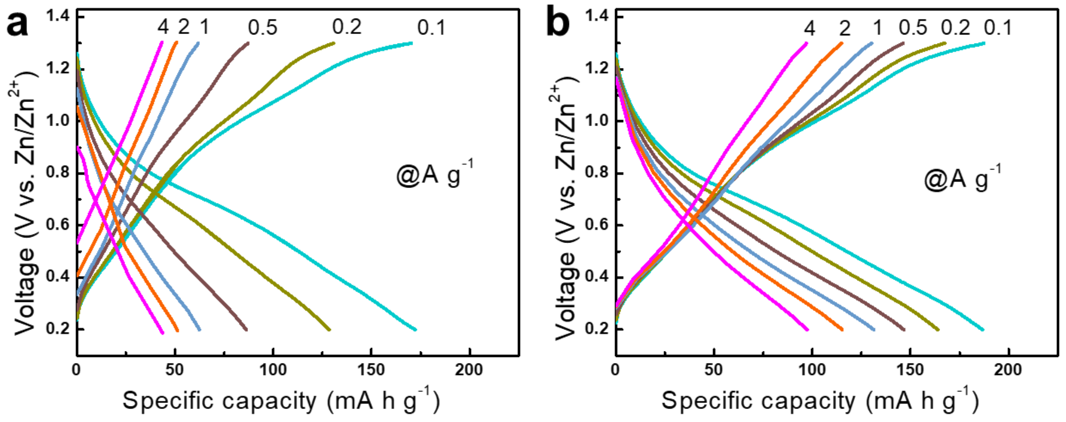

Figure S21 Charge-discharge profiles of (a) bare Zn/MoS_2 and (b) $CeO_2@Zn/MoS_2$ full cells at different current densities.

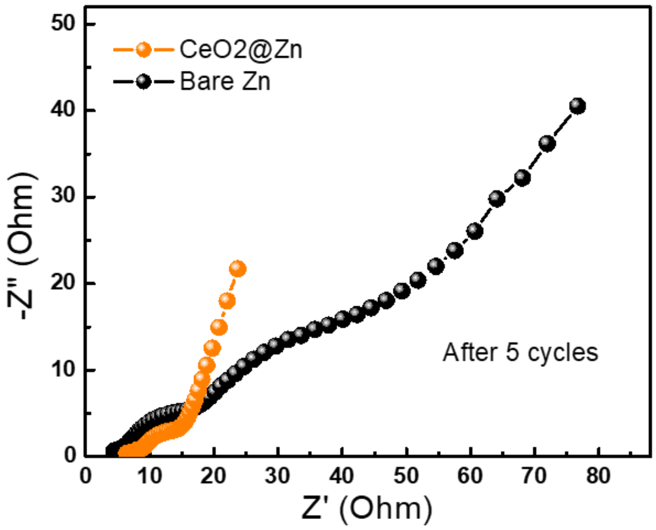

Figure S22 EIS plots of bare Zn/MoS_2 and CeO_2@Zn/MoS_2 full cells after 5 cycles.
